# Generalizing machine learning models from clinical free text

**DOI:** 10.1038/s41598-025-17197-6

**Published:** 2025-08-28

**Authors:** Balaji Pandian, John Vandervest, Graciela Mentz, Jomy Varghese, Shavano D. Steadman, Sachin Kheterpal, Maggie Makar, V. G. Vinod Vydiswaran, Michael L. Burns

**Affiliations:** 1 Department of Anesthesiology, Weill Cornell Medicine, New York, NY USA; 2 Department of Anesthesiology, University of Michigan, 1500 East Medical Center Drive, 1H247 UH, SPC 5048, Ann Arbor, MI 48109-5048 USA; 3 Department of Urology, University of Michigan, Ann Arbor, MI USA; 4 Division of Computer Science and Engineering, University of Michigan, Ann Arbor, MI USA; 5 Department of Learning Health Sciences, University of Michigan, Ann Arbor, MI USA; 6 School of Information, University of Michigan, Ann Arbor, MI USA

**Keywords:** Health care, Medical research, Outcomes research, Computer science

## Abstract

To assess strategies for enhancing the generalizability of healthcare artificial intelligence models, we analyzed the impact of preprocessing approaches applied to medical free text, compared single- versus multiple-institution data models, and evaluated data divergence metrics. From 1,607,393 procedures across 44 U.S. institutions, deep neural network models were created to classify anesthesiology Current Procedural Terminology codes from medical free text. Three levels of text preprocessing were analyzed from minimal to automated (cSpell) with comprehensive physician review. Kullback–Leibler Divergence and k-medoid clustering were used to predict single- vs multiple-institutional model performances. Single-institution models showed a mean accuracy of 92.5% [2.8% SD] and 0.923 [0.029] F1 on internal data but generalized poorly on external data (− 22.4% [7.0%]; − 0.223 [0.081]). Free text preprocessing minimally altered performance (+ 0.51% [2.23]; + 0.004 [0.020]). An all-institution model performed worse on internal data (-4.88% [2.43%]; − 0.045 [0.020]), but improved generalizability to external data (+ 17.1% [8.7%]; + 0.182 [0.073]). Compared to vocabulary overlap and Jaccard similarity, Kullback–Leibler Divergence correlated with model performance (R^2^ of 0.41 vs 0.16 vs 0.08, respectively) and was successful clustering institutions and identifying outlier data. Overall, pre-processing medical free text showed limited utility improving generalization of machine learning models, single institution models performed best but generalized poorly, while combined data models improved generalization but never achieved performance of single-institutional models. Kullback–Leibler Divergence provided valuable insight as a reliable heuristic to evaluate generalizability. These results have important implications in developing broad use artificial intelligence healthcare applications, providing valuable insight into their development and evaluations.

## Introduction

Artificial intelligence (AI) medical applications have rapidly expanded over the past decade, however, medical AI has suffered repeated setbacks due to an inability to demonstrate wide clinical use^1^, particularly beyond training datasets^2^. Adapting to novel data, known as generalizability, remains a major challenge. Success in clinical practice relies on understanding and enhancing the generalizability of AI models, an area currently lacking sufficient knowledge^3^. Classification of anesthesiology Current Procedural Terminology (CPT) codes from procedural clinical free text presents an opportunity to study generalizability of medical machine learning (ML) models as assignment is a single label selected from a large but finite solution space (< 300 codes). Assignment requires complex interpretation of medical procedural free text, and while there have been past successes in model development^4,5^, generalizability has not been extensively studied.

Access to healthcare data is limited given privacy concerns, however public–private partnerships to support nationwide health AI assurance labs to test clinical AI models are underway^6^ and would benefit from investigation into generalizability. Techniques like hold-out, cross-validation, and bootstrapping^7^ minimally predict generalizability, while automated processing and human review have variable success^8^. Medical free text is a common and rich source of training data for models, but documentation is prone to variations in clinician writing styles, grammar, and vocabulary. Spelling, translational, grammatical, and copy-forward error rates are as high as 10% in medical text^9^ and can significantly reduce ML model performance^10,11^. Preprocessing data can enhance performance^12^. Natural Language Processing (NLP) can recognize and correct errors^13–16^ and there exist NLP software tailored for medical applications^9^, including the Unified Medical Language System (UMLS)^17^ (biomedical vocabularies including words, abbreviations, and expansions) and CSpell^18^ (health inquiry spell-checker). Separately, comparative analysis of datasets offers a means to identify ML model generalizability. The Kullback–Leibler divergence (KLD) is a statistical measure of divergence between probability distributions^19^ which has improved ML classifications^20,21^, and may be useful in determining model generalizability to new datasets before model testing.

Our study presents a multicenter investigation of ML model generalizability. Employing data from forty-four distinct institutions from a national anesthesiology registry, deep neural network (DNN) models were developed to classify anesthesiology CPT codes. The main contributions of this manuscript are as follows:

- Investigate preprocessing medical free text using expert-levels of feature engineering preprocessing compared to DNN learned representations.
- Generalizability assessments using models trained from forty-four independent healthcare institutions, using both single institutional and large multicenter aggregated models.
- Investigation of KLD correlation to external model performance, for potential as a heuristic to evaluate ML generalizability.

## Methods

### Study design

Due to the retrospective nature of the study, The University of Michigan Institutional Review Board waived the need of obtaining informed consent. All experiments were performed in accordance with relevant guidelines and regulations, including STROBE guidelines^31^. Data was obtained from the Multicenter Perioperative Outcomes Group (MPOG) database [http://mpog.org], a registry consisting of procedures requiring anesthesia from hospitals across the United States^32^. The study protocol was approved by the MPOG research committee prior to accessing the data. Data was collected from all procedures (adult and pediatric patients) between January 1st, 2017, and July 31st, 2019. Procedures with missing or incomplete text or billing data were excluded. Procedures were limited to those with anesthesiology CPT codes shared among all institutions.

### Machine learning model creation

Supervised ML models were created to classify anesthesiology CPT codes from procedural text: preprocessed text strings were vectorized using a term frequency-inverse document frequency (TF-IDF) matrix and input into a DNN. The entire procedural text was used without truncation. Categorical cross-entropy was used as the loss function. We used Adam to optimize weights^33^. Additionally, a 25% dropout layer was added between each layer. The network architecture is fully depicted in Figure S1. This specific architecture was experimentally determined as optimal after testing various neural network configurations varying between 2 to 6 layers, dropout of 0 to 50%, and various layer sizes from {2000, 1000, 500, 250, 200, 150, 100, 50}. The chosen network architecture performed optimally on various single institution training and separate institution testing. A three layer neural network with dimensions 500 (RELU activation, 25% dropout regularization), 250 (RELU, 25% dropout), and 48 (softmax) was experimentally determined. Models were created and evaluated using a NVidia Titan RTX GPU, Python v3.9.7, Tensorflow v2.6.0, and CUDA v11.2.

For each single institution, an ML model was trained using the entire institution-specific data from the analytical dataset, then independently evaluated on the entire data from every other single institution. This process was repeated for every pair of institutions at each preprocessing level. Results were then averaged across all individual institutions, omitting self evaluations unless explicitly stated. Two types of combined machine learning models were created. “80:20” was a single model, created on a combined 80% and tested on the remaining combined 20% of data from each institution. “Holdout” models were a series of models created by training on data from all but one institution, using the remaining single institution as a holdout. For combined (multicenter) models, five-fold cross validation was used to ensure no overlap of data between training and testing. Individual institutional models were trained on a single institution with each other institution acting as true holdouts for testing.

### Model performance metrics

Model accuracy and F1-scoring (all micro-averaged unless otherwise specified) were used as evaluation metrics for comparing predicted to documented institution-billed anesthesiology CPT charges (again, limited to those CPT codes present in all institutions). Pairwise evaluation was conducted in which a model was trained on one or more institutions and evaluated on data from one or more institutions. Five-fold cross validation was used to ensure no overlap of data between training and testing.

### Clinical free text preprocessing

Institutional vocabulary is defined as all the unique words used in procedural text at a specified institution. Vocabularies were compared before and after text preprocessing, with vocabulary overlap between any two institutions defined as the (number of unique words used from institution A that appear in institution B) divided by (number of unique words used from institution B). This non-symmetric overlap methodology was chosen to mimic an institution training a model on its own data and transferring that model to other institutions. Traditional symmetric Jaccard Similarity was also evaluated across institution pairs.

Model features consisted of clinical procedural free text, a word string describing the procedure. Three levels of text preprocessing were investigated: “Minimal,” “cSpell,” and “Maximal.” “Minimal” preprocessing included removing stop words and punctuations, trimming multiple consecutive, leading, and trailing white space, lowercasing, and converting numbers from 1–10 to words. “cSpell” preprocessing included all preprocessing from “Minimal” plus programmatic identification of misspelled words by the UMLS cSpell tool. “Maximal” preprocessing included “cSpell” preprocessing plus manual expansion of acronyms and misspelled words by physician experts: misspelled and acronym text strings were identified by programmatic cross-referencing against the UMLS Specialist Lexicon tool and identified strings manually corrected and/or expanded by two physician authors with domain knowledge in anesthesiology billing codes (BP and MB). Inter-rater reliability (kappa statistic, a statistical test of symmetry) was used to assess the two physician experts.

### Kullback–Leibler divergence

KLD is a well-accepted measure of divergence between two probability distributions. KLD metrics were created evaluating distribution of CPT codes (model labels, “KLD_CPT”) and distribution of unique individual words within a procedural text for each CPT (model features, “KLD_word”) between every pain of institutions. Additionally, a “composite KLD” was created for each institutional pair that normalizes each KLD_word by CPT difference between two institutions (Equation S1). These metrics describe the variation between institutions for: (1) procedural diversity (CPT distribution) and (2) clinical text used to describe identical procedures (difference between institutions in procedural text for the same CPT code). Higher KLD values correspond to greater dissimilarity between datasets being compared, with a KLD value of 0 indicating identical datasets. The Pearson product correlation was assessed across all KLD and F1-score pairwise measurements between institutions.

### Institutional clustering

Data was clustered on composite KLD using k-medoid clustering via the PyClustering Python library^34^. Multidimensional scaling was used to plot the clusters of institutions on a cartesian plane^35,36^. Number of centroids(k) was determined by incrementing k from 2 to 25 until optimal clustering was achieved using the Elbow Method and the sum of squared distances, resulting in a k of 5.

## Results

### Clinical free text preprocessing and institutional pairwise analyses

The final dataset consisted of 1,607,393 procedures: 44 institutions covering 48 distinct anesthesiology CPT codes (From 3,566,343 procedures, 432,039 were removed due to missing data and 1,526,911 containing CPT codes not shared between all institutions, Figure S2). Procedures were majority women (866,284; 53.9%) aged 49.3 [22.4] years, mean [SD]. Using the American Society of Anesthesia Physical Status Classification Score (ASA), a clinically accepted measure of patient severity, most were ASA 2 (693,590; 43.1%) or ASA 3 (629,978; 39.2%). Procedures on pediatric patients (190,353 or 11.8%), ASA emergent status (88,432 or 5.5%), and the following three most common anesthesia CPT codes: 00840 (141,538, 8.8%), 00790 (126,691, 7.9%), and 00400 (119,026, 7.4%) were found (Table S1).

**Fig. 1:**
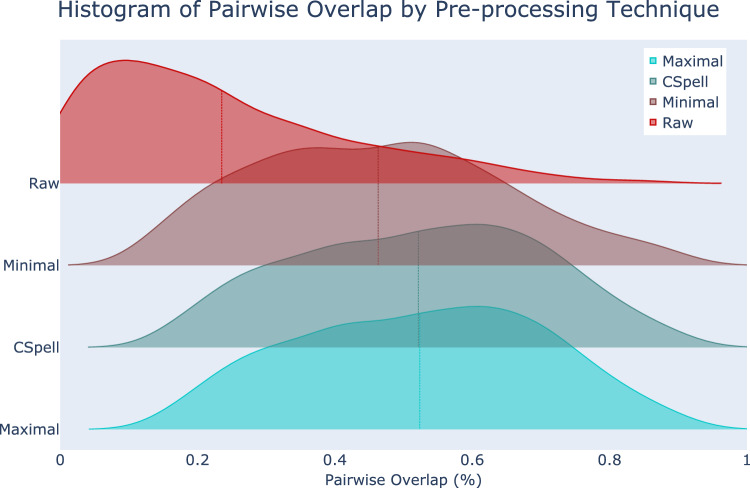
Composite histogram of individual pairwise vocabulary overlap with various methods of text preprocessing. Each preprocessing technique represents 1892 pairwise comparisons (*institution a* vocabulary in *institution b* where *a* ! = *b*). Vocabulary reduction and overlap are improved with each preprocessing techniques between individual institutions.

Table 1 shows examples of procedural text. Preprocessing consisted of one of three techniques: Minimal, CSpell, and Maximal, described in methods. Maximal preprocessing used manual expansion of acronyms and misspelled words from two independent physicians. Of 4902 manually corrected strings, 120 were co-evaluated showing a simple kappa inter-rater reliability of 0.9238 (0.8643–0.9834 95% confidence). There were 216,763 unique terms used across all surgical procedure texts. Minimal preprocessing reduced vocabulary to 64,249 (30.0% of original) while cSpell and Maximal reduced to 37,371 (17.2%) and 37,098 (17.1%). Without preprocessing, average vocabulary overlap between two institutions was 23.5% [18.3% SD, 0.1–87.7% min–max]. Most pairings (89.1%, 1686/1892) showed < 50% overlap (Figure S3). With minimal preprocessing, average vocabulary overlap increased to 46.3% [18.2% SD, 9.7–92.1%], cSpell 52.2% [17.9%, 12.5–92.7%], and Maximal 52.4% [17.9%, 12.6–92.6%] (Fig. 1). A similar increase in Jaccard Similarity is demonstrated between institutions from minimal to cSpell to Maximal Pre-processing (Figure S4). Approximately 10% of words were identified misspelled by CSpell while 9% of procedures contained text with at least one misspelling.

**Table 1:** Procedural Text and Anesthesiology CPT Examples.

Procedural text	Anesthesiology CPT code	Institution
Left total knee replacement	01402	44
Left total knee revision, with application of prevena incision management system	01402	44
Bilateral knee revision, total knee arthroplasty; 1 component *** poly exchange—bilateral ;bilateral arthroplasty, femoral condyles/tibial plateau(s), knee; with debridement & partial synovectomy	01402	44
27,447, Total Knee Replacement, Right, Knee	01402	37
Robotic-assisted total replacement of right knee R TKA (arthroscopy repalcement total knee) (right knee)	01402	16
Total replacement of left knee (arthroplasty repalcemnt total knee) (left knee)	01402	16
Right total knee arthroplasty (right knee)	01402	16

Examples of actual, unprocessed, procedural text (model features) and Anesthesiology CPT code billed by the institution (labels) for common knee procedures comprising the 01402 CPT code from multiple institutions. Misspellings and capitalization are reproduced exactly from the data. Institution variations in the text corresponding to the same CPT highlight the challenges inherent to training models that generalize. *CPT* Current Procedural Terminology.

**Fig. 2:**
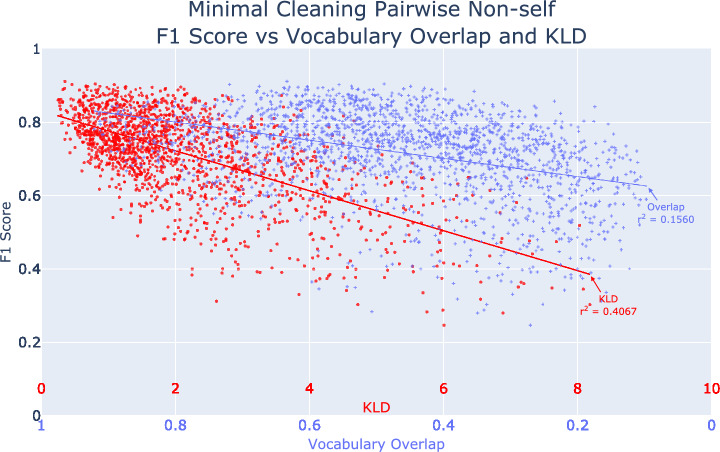
F1-score vs. overlap and KLD. Each point represents an individual pair of non-self institutions. Blue dots are compared against overlap while red dots are compared against KLD. The Minimal data preprocessing level was used in modeling.

Institutional models were evaluated on every institution using the three preprocessing methods, resulting in 132 models and 5,676 pairwise evaluations. Using minimal preprocessing, average self-institution accuracy was 92.5% [2.8% SD] with 0.923 [0.029] F1 (Table 2). Average decrease in accuracy and F1-score from self to non-self data was 22.4% [7.0%] and 0.223 [0.081]. Preprocessing improved performance (Minimal to Maximal preprocessing: + 0.51% [2.23%] accuracy; + 0.004 [0.020] F1-score). Vocabulary overlap weakly correlated with model accuracy and F1-score (average R^2^ 0.16, Fig. 2). Jaccard Similarity was not only weakly correlated with model accuracy, but also performed worse than vocabulary overlap (average R^2^ 0.08, Figure S5).

**Table 2:** Results of models trained on the output of various preprocessing techniques.

	Non-Self		Self	
Preprocessing Method	Accuracy, Average [SD]	F1 Score, Average [SD]	Accuracy, Average [SD]	F1 Score, Average [SD]
Minimal	71.9% [10.4%]	0.717 [0.114]	92.5% [2.8%]	0.923 [0.029]
cSpell	72.1% [10.3%]	0.719 [0.114]	92.3% [2.9%]	0.921 [0.030]
Maximal	72.2% [10.2%]	0.721 [0.112]	92.3% [2.9%]	0.923 [0.029]

Average Improvement	Accuracy, Average [SD]	F1 Score, Average [SD]	Accuracy, Average [SD]	F1 Score, Average [SD]
Maximal – Minimal	+ 0.51% [2.23%]	+ 0.004 [0.020]	− 0.23% [0.31%]	− 0.002 [0.004]
Maximal – cSpell	+ 0.30% [2.19%]	+ 0.003 [0.020]	− 0.04% [0.20%]	− 0.002 [0.003]
cSpell – Minimal	+ 0.20% [1.25%]	+ 0.001 [0.012]	− 0.20% [0.40%]	0.000 [0.002]

There existed minimal difference between model performances from different preprocessing techniques. “Non-self” designates models trained on a single institution and repeatedly tested on each separate (non-self) institution. “Self” represents models trained on a single institution and tested on the same institution. Three levels of text preprocessing were investigated: “Minimal,” “cSpell,” and “Maximal,” as described in Methods, Clinical Free Text Preprocessing.

### Models from combined institutional data

The “80:20” combined data model yielded an average [SD] accuracy of 87.7% [4.0%] and F1-score of 0.879 [0.386]. Compared to individual models tested on self-data, the 80:20 model performed worse for each institution, yielding 4.88% [2.43%] less accuracy and 0.045 [0.020] less F1-score. For non-self data, the model performed better in every instance, yielding 17.1% [8.7%] higher accuracy and 0.182 [0.073] higher F1-score. Forty-four “holdout” models yielded an average accuracy of 84.6% [5.1%] and 0.848 [0.491] F1-score. Compared to individual models tested on self-data, holdout models performed worse on every comparison, (8.01% [3.72%] less accuracy and 0.075 [0.034] less F1-score). For non-self data, the holdout models performed better in every instance, increasing accuracy an average of 17.1% [8.7%] and F1-score 0.167 [0.073].

### Kullback–Leibler divergence

KLD metrics were created for individual word, CPT, and composite (KLD_word, KLD_CPT, and KLD_Composite) for each institutional pair. Compared to vocabulary overlap, all KLD metrics showed improved correlation in accuracy and F1-scoring (Fig. 2, Figure S6), with composite KLD showing an R^2^ of 0.41. As KLD values increased (less similar data distributions) model performance worsened. Vocabulary overlap alone yielded poor correlation with an R2 of 0.15–0.16 (Figure S6). KLD_word showed an R^2^ of 0.23 and KLD_CPT an R^2^ of 0.33. The average Pearson product-moment correlation for composite KLD was − 0.8127 (Min: − 0.882, Max: − 0.644) with all institutional pairs exhibiting strong negative correlation; whereas pairwise vocabulary overlap again yielded a weaker negative correlation of − 0.4423 (Min: − 0.674, Max: 0.0091).

### Institutional clustering

**Fig. 3:**
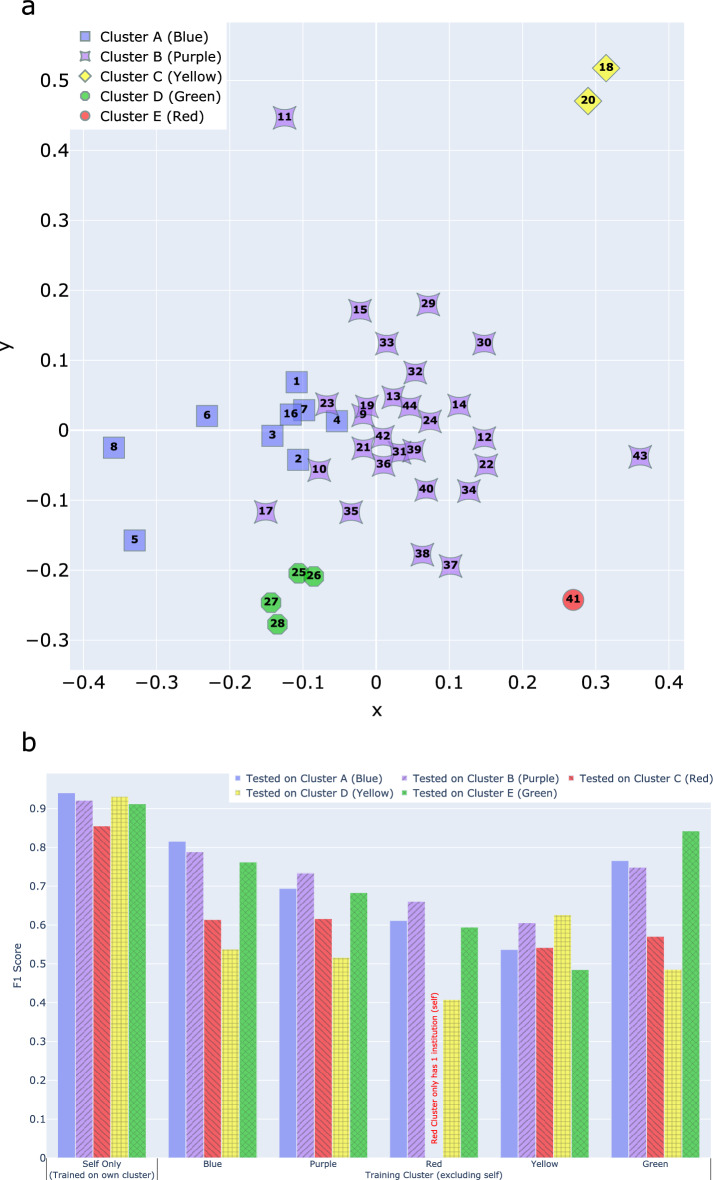
(**a**) 2-Dimensional cartesian plane representation of clustering showing five non-overlapping unbalanced clusters derived from k-medoid clustering using composite KLD. (**b**) F1-score by testing clustering group. Self only is self-institution testing. Scores are reported as averages. The Minimal data preprocessing level was used in modeling.

Composite KLD was used for k-medoid clustering, resulting in nine blue cluster institutions, twenty-eight purple, one red, two yellow, and four green (Fig. 3a). Testing on non-self institutions, models performed better on intracluster vs. intercluster data: blue (81.6% [6.8%] accuracy [SD] vs. 77.0% [6.6%]), purple (73.4% [8.1%] vs 66.9% [9.5%]), green (83.9% [1.6%] vs. 74.5% [6.8%]), yellow (61.0% [8.8%] vs. 56.8% [6.6%]). Intracluster analysis was not possible for red (single institution) but showed 62.8% [5.9%] intercluster accuracy. For F1-scoring: blue (0.816 [0.072] vs. 0.767 [0.077]), purple (0.734 [0.092] vs 0.664 [0.105]), green (0.842 [0.015] vs. 0.735 [0.084]), yellow (0.627 [0.092] vs. 0.578 [0.081]), and red (N/A vs. 0.633 [0.105]) (Fig. 3b).

The single red cluster institution generalized poorly (average accuracy and F1-score of 61.4% [10.9%] and 0.608 [0.110], compared to 72.2% [7.2%] and 0.720 [0.087] for all other institutional combinations). Likewise, institutions 11, 37, 38, and 43 performed relatively poorly compared to purple peers (intracluster accuracy and F1-score of 62.9% [9.6%] and 0.594 [0.118] compared to 75.2% [6.3%] and 0.757 [0.062]). Composite KLD values deviated from the cluster, 2.2 [0.9] vs 1.2 [0.5]. Comparing institutional demographics (Table S1), institution 11 data contained 93.1% of procedures on pediatric patients vs. a dataset average of 11.8%. Outliers also showed greater proportions of ASA 1 patients and lower percent of major surgical procedures.

## Discussion

Generalizing ML models across diverse healthcare systems remains challenging. In this study, we explored individual and combined institutional ML models evaluated across 44 distinct healthcare institutions. We showed that (1) generalizing machine learning models to external institutions without training on external data yielded poor results, (2) feature (medical free text) normalization through non-learned traditional pre-processing did not significantly improve performance with DNN models, (3) models using data from multiple institutions greatly improved external performance but internal performance never reached the level of single-institutional models, and (4) KLD comparison of model features and labels correlated with model results and could be an early heuristic for model performance. These results are important considerations for individual institutions and nationwide health AI assurance labs created to evaluate clinical AI models.

Vocabulary overlap of the individual words used in procedural text (model features) was low between institutions. Surprisingly, improving vocabulary overlap using increasing levels of preprocessing (Minimal—> cSpell—> Maximal) yielded insignificant gains in model accuracy and F1-scores. Although these techniques were successful in removing linguistic noise and normalizing the input text (total vocabulary size reduced by ~ 5 × and pairwise overlap increased by 28.9%), these results suggest extensive preprocessing may be unnecessary for AI applications using medical free text. Tasks such as clinical abbreviation disambiguation are less crucial, while resource-intensive manual review efforts, such as physician-lead text adjudication, may be costly and unnecessary. DNNs were selected for ML models due to their ubiquitous utility; and chosen over alternatives, such as recurrent neural networks and larger transformer language models, due to the short length of procedural text features and previous demonstrations of equality in CPT classification tasks between these model types^4,22^. The study results could mean DNN models may be adept at improving text differences internally, decreasing the effect of text normalization^23,24^, though further investigation is warranted. Preprocessing medical free text has historically had mixed effects on model performance. Correcting errors in free text clinical documents^25^ only modestly increased F-measurement^26^, while preprocessing techniques for International Classification of Diseases code classification yielded only minor improvements as models were able to pick up specific language use such as abbreviations and synonyms^12^.

Large, multicenter models have historically generalized suboptimally, as was evident following wide adoption of the Epic proprietary sepsis prediction model, where the multicenter model performed poorly on individual sites (0.63 local AUC vs 0.76–0.83 vendor reported AUC)^27^. In our study, the 80:20 and the holdout models outperformed all single-institution models when evaluating external data, but underperformed on internal data, again highlighting the need for training or tuning ML models on local data. The combined training approach is appealing for its simplicity, but as data expands and becomes increasingly skewed there are increased requirements in model development and optimal retraining.

KL divergence is a powerful tool for comparing probability distributions due to its strong theoretical foundation, its usefulness in various machine learning tasks, and its relative simplicity compared to other metrics like Jensen–Shannon divergence or Wasserstein distance. While these other metrics have their own strengths, KL divergence remains a valuable choice for many applications due to its versatility and the insights it provides into information loss and approximation. KLD measures of data distributions correlated with model evaluations, acting as a potential a priori measure of model performance. Word (feature), CPT (label), and composite KLD measurements each correlated better than vocabulary overlap, displaying the importance and promise of using data distribution statistics as heuristics for machine learning model performance on new or changing datasets. Utilization of these distribution techniques could determine when new model creation vs retraining/tuning is appropriate. Similar studies have used semantic similarity to estimate model portability, comparing cohorts by word-similarity scoring methods^28^. From their results, the authors were able to identify variations and assertion differences between cohorts and recover 0.09 of the initial 0.13 loss in F-score from the external dataset. As models expand use beyond training datasets, it is imperative to assess applicability prior to implementation. Investigating the differences in model features and labels in new datasets is a logical step in a priori generalizability determination^19,29^.

KLD also proved useful in discovering outliers where model performance significantly lagged. K-medoid clustering of composite KLD yielded five unbalanced institutional clusters. The red cluster consisted of a single institution which performed poorly on all other institutional data and is a relative outlier. Four additional outliers were discovered within the purple cluster. Models from these outlier institutions performed poor relative to peer institutions generalizing beyond their own data. These clusters represent semantic and/or distributive similarity. Demographic data from outlier institutions identify potential deviations in distributions, such as pediatric and outpatient focuses. These outlier institutions perform better with institution-specific models and serve as examples for using divergence metrics to help determine local vs. combined model development.

Limitations in this study include: (1) models trained on billing data contain inherent manual billing errors. Manually-assigned anesthesiology CPT codes were used as gold standards. Individual institutions uploaded billing data, including anesthesiology CPT codes, directly to the MPOG coordinating center. MPOG anesthesiology CPT codes were previously evaluated by manual review and determined to contain a modest 5% error rate^4^. (2) While previous studies using different modeling architectures have proven non-superior, including long short-term memory and transformer models, a single machine learning model type, DNN, was used throughout this study and may not extend to other model types^3,4^. (3) Large language models were not explored due to practical limitations related to security, computational cost, and deployment feasibility. While large language models are promising, there are challenges associated with training, fine-tuning, and maintaining these models, whether developed in-house or accessing commercial vendors. Although further work is certainly warranted, as a result, our focus remained on using accessible and broadly implementable approaches. (4) Composite KLD was used as a divergence metric by multiplying individual KLD divergence scores: feature (word) by label (CPT). There are alternative ways to produce a composite score which merit further investigation. (5) While TF-IDF vectorization was chosen for simplicity and equivalent results relative to embedding techniques, pretrained language models using contextual or latent space embeddings, such as ClinicalBERT^30^, are advanced methods that may lead to improved performance and should be investigated.

## Supplementary Information

Supplementary Information.

## Data Availability

The datasets generated and/or analyzed during the current study are not publicly available due as the datasets involved in this study are defined as limited datasets per United States Federal Regulations and require execution of a data use agreement for transfer or use of the data. They are derived from data shared within the Multicenter Perioperative Outcomes Group (MPOG). The investigative team is able to share data securely and transparently on reasonable request to the corresponding author, conditional on: (i) receipt of a detailed written request identifying the requestor, purpose and proposed use of the shared data, (ii) use of a secure enclave for the sharing of personally identifiable information and (iii) the request is permissible within the confines of existing data use agreements executed between MPOG members.
