# Supplementary material for: Generalizing machine learning models from clinical free text
Source: Sci Rep. 2025 Aug 28;15:31668. doi: 10.1038/s41598-025-17197-6 (PMC12391454; doi:10.1038/s41598-025-17197-6)
Supplement: Supplementary file 1 — Supplementary Information. [file 41598_2025_17197_MOESM1_ESM.docx]

**Electronic Supplemental Materials**

**Table of Contents**

**Figure S1:** Complete neural network model architecture

**Figure S2:** Study Flowchart

**Table S1:** Study Demographics

**Equation S1:** Creation of the Kullback-Leibler Divergence (KLD) Composite Score

**Figure S3:** Pairwise heatmap of vocabulary overlap between all institutions

**Figure S4:** Composite histogram of Jaccard Similarity between institutions

**Figure S5:** Pairwise F1 score vs. Jaccard Similarity and KLD

**Figure S6:** Accuracy and F1 score vs. vocabulary overlap and KLD


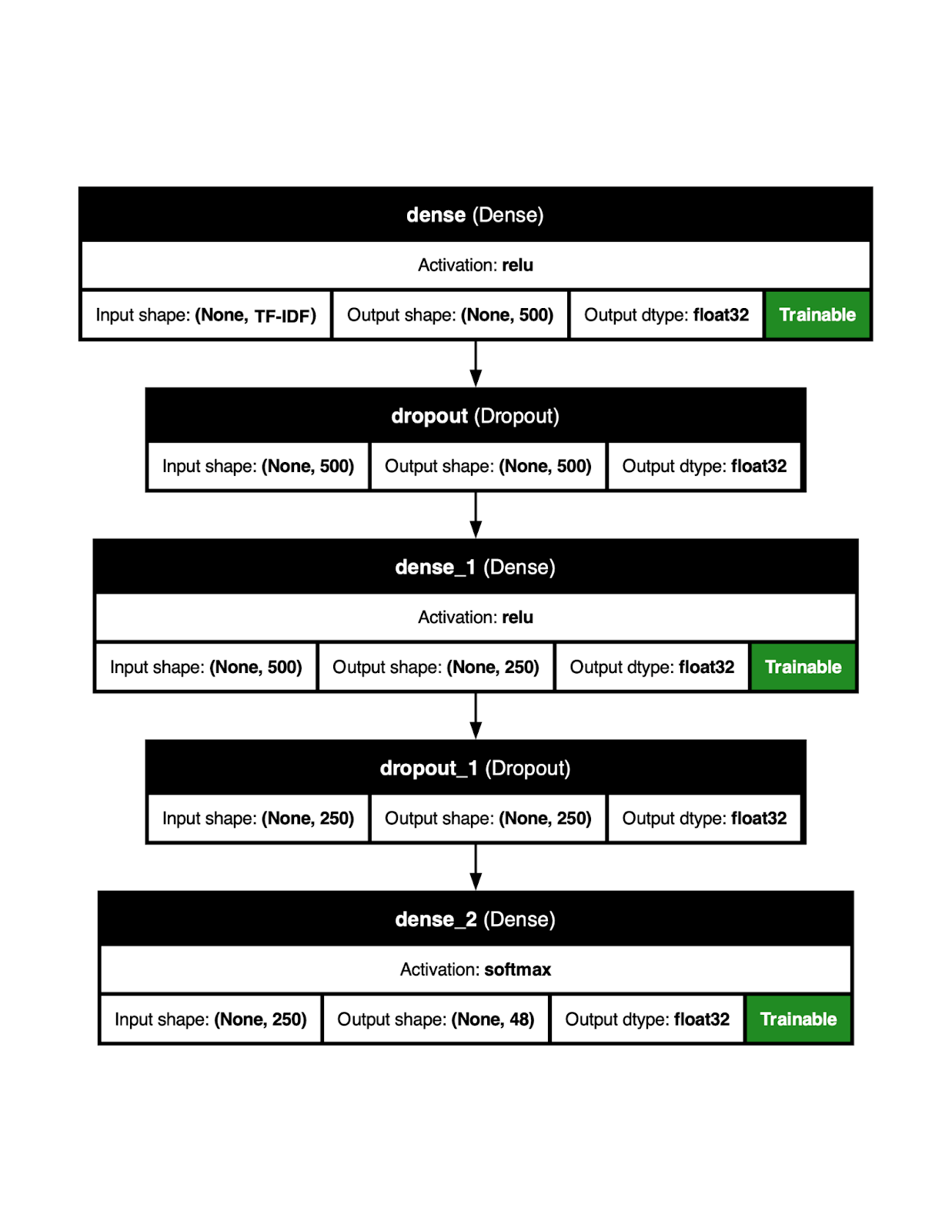


**Figure S1:** Complete neural network model architecture. The input layer size for the first layer (marked as “TF-IDF”) varied depending on the input size of the TF-IDF vectorization for each created model.


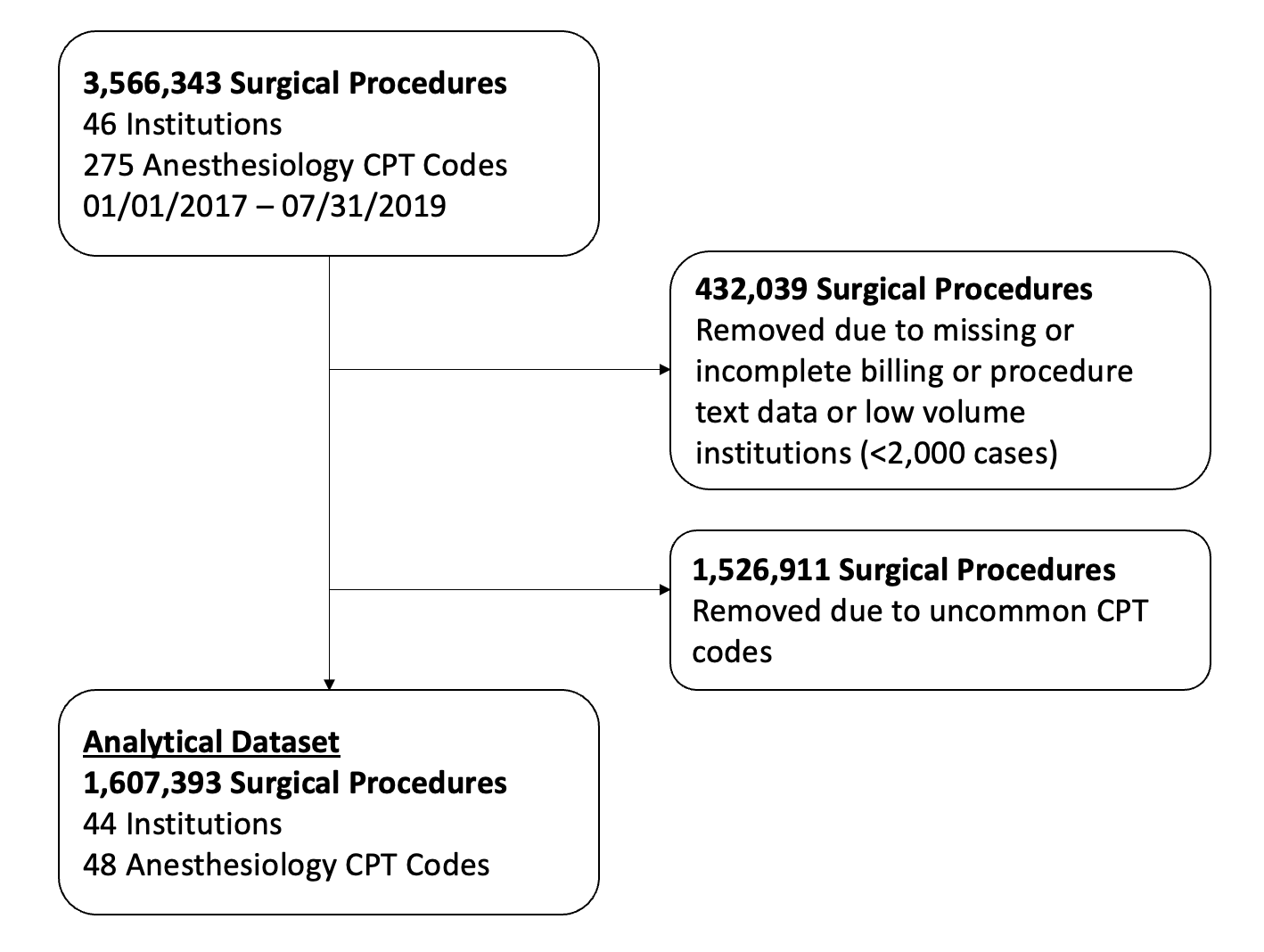


**Figure S2:** Study Flowchart


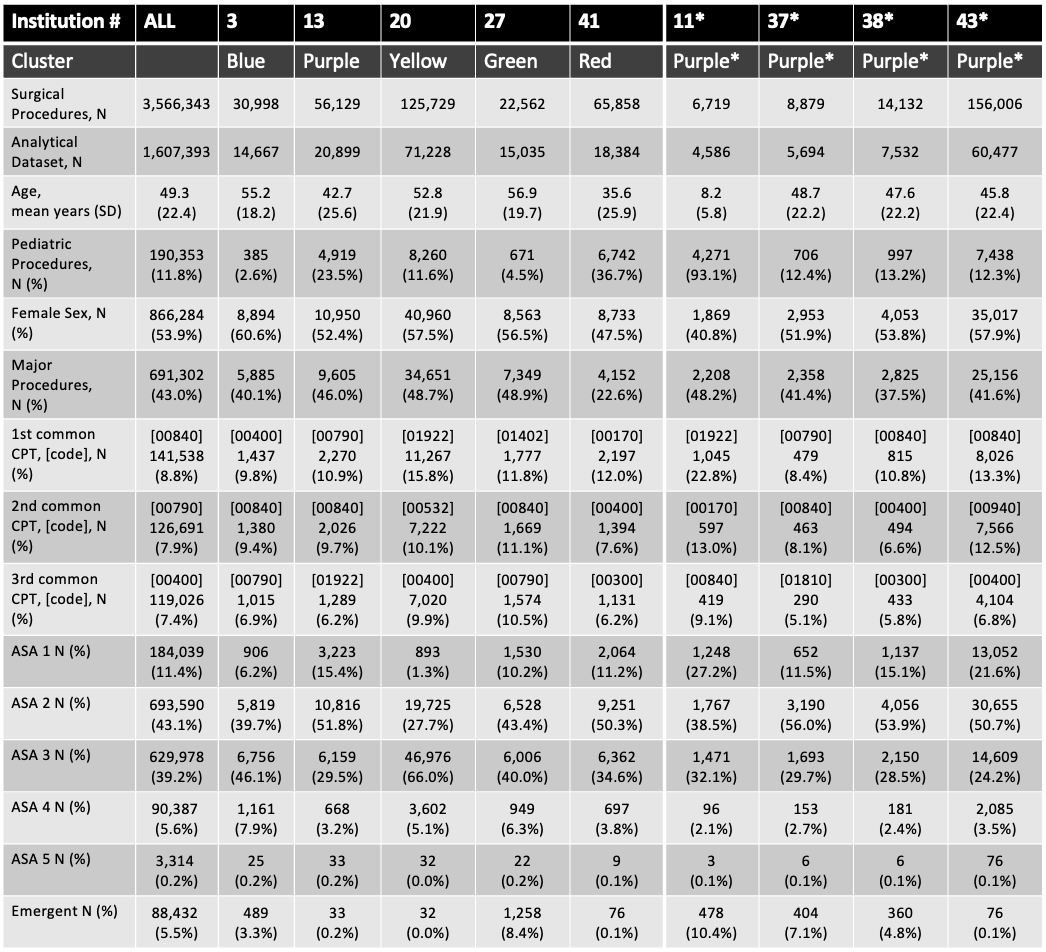


**Table S1:** Study Demographics for all surgical procedures in the analytical dataset. Pediatric procedures are defined as patient age <18.0 years old at the time of the surgical procedure. Percentages are respective to each column. Additional purple cluster institutions (*) are explicitly included for sub-cluster analysis. Notably, Institution 11 represents a primarily pediatric institution.

**Equation S1: Creation of the Kullback-Leibler Divergence (KLD) Composite Score**

For each institution pair, a composite Kullback-Leibler divergence (KLD) metric was created to evaluate both the distribution of Current Procedural Terminology (CPT) codes and the distribution of unique individual words within a procedural text for each CPT. These individual KLD values were combined (product) into a single composite for each institutional pair, such that:

$$KLD_{CPT\left( inst_{a}, inst_{b} \right)}=KLD\left( \left( \frac{CASECOUNT\left( inst_{a},c \right)}{CASECOUNT\left( inst_{a}, C \right)} \right), \left( \frac{CASECOUNT\left( inst_{b}, c \right)}{CASECOUNT\left( inst_{b}, C \right)} \right) | c\in C \right)$$

$${KLD}_{word\left( inst_{a}, inst_{b} \right)} =\sum_{cpt \in C}^{cpt} KLD\left( \left( \frac{WORDCOUNT(inst_{a}, w, cpt)}{CASECOUNT(inst_{a}, cpt)} \right), \left( \frac{WORDCOUNT(inst_{b}, w, cpt)}{CASECOUNT(inst_{b}, cpt)} \right)|w\in WORDS((inst_{a}\cup inst_{b}),cpt) \right)$$

$${KLD}_{composite(inst_{a}, inst_{b})}=\sum_{cpt \in C}^{cpt} \left[ Component(1)*Component(2) \right]$$

$$Component(1) =ABS\left( \frac{CASECOUNT(inst_{a}, cpt)}{CASECOUNT(inst_{a}, C)}-\frac{CASECOUNT(inst_{b}, cpt)}{CASECOUNT(inst_{b}, C)} \right)$$

$$Component(2) =KLD\left( \left( \frac{WORDCOUNT(inst_{a}, w, cpt)}{CASECOUNT(inst_{a}, cpt)} \right), \left( \frac{WORDCOUNT(inst_{b}, w, cpt)}{CASECOUNT(inst_{b}, cpt)} \right)| w \in WORDS((inst_{a}\cup inst_{b}),cpt) \right)$$

Given:

1. $inst_{a}\in\{44 institutions\}, inst_{b}\in\{44 institutions\}, C =\{48 CPT codes\}$
2. $ABS(x) = absolute value of x$
3. $KLD\left( p\left( w \right), q\left( w \right) \right)= KL Divergence between distribution p and q$
4. $CASECOUNT(i, c) = number of cases with CPT (c) in institution (i)$
5. $WORDCOUNT(i, w, c) = count of instances of word (w) in cases with CPT (c) in institution (i)$
6. $WORDS(i,c) = unique words in institution (i) with CPT (c)$


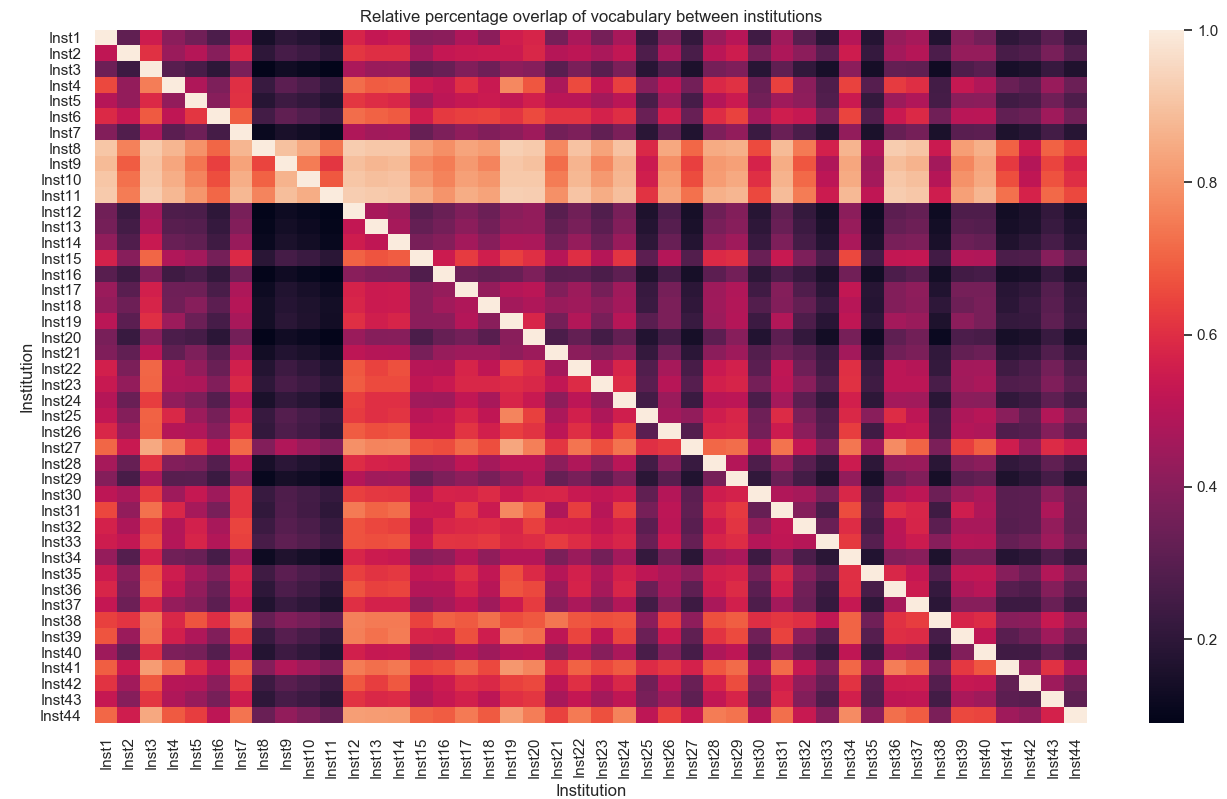


**Figure S3:** Pairwise heatmap of vocabulary overlap between all institutions, without text preprocessing. Percentage / fraction of vocabulary overlap of the x-axis institution into the y-axis institution. Inst = institution.


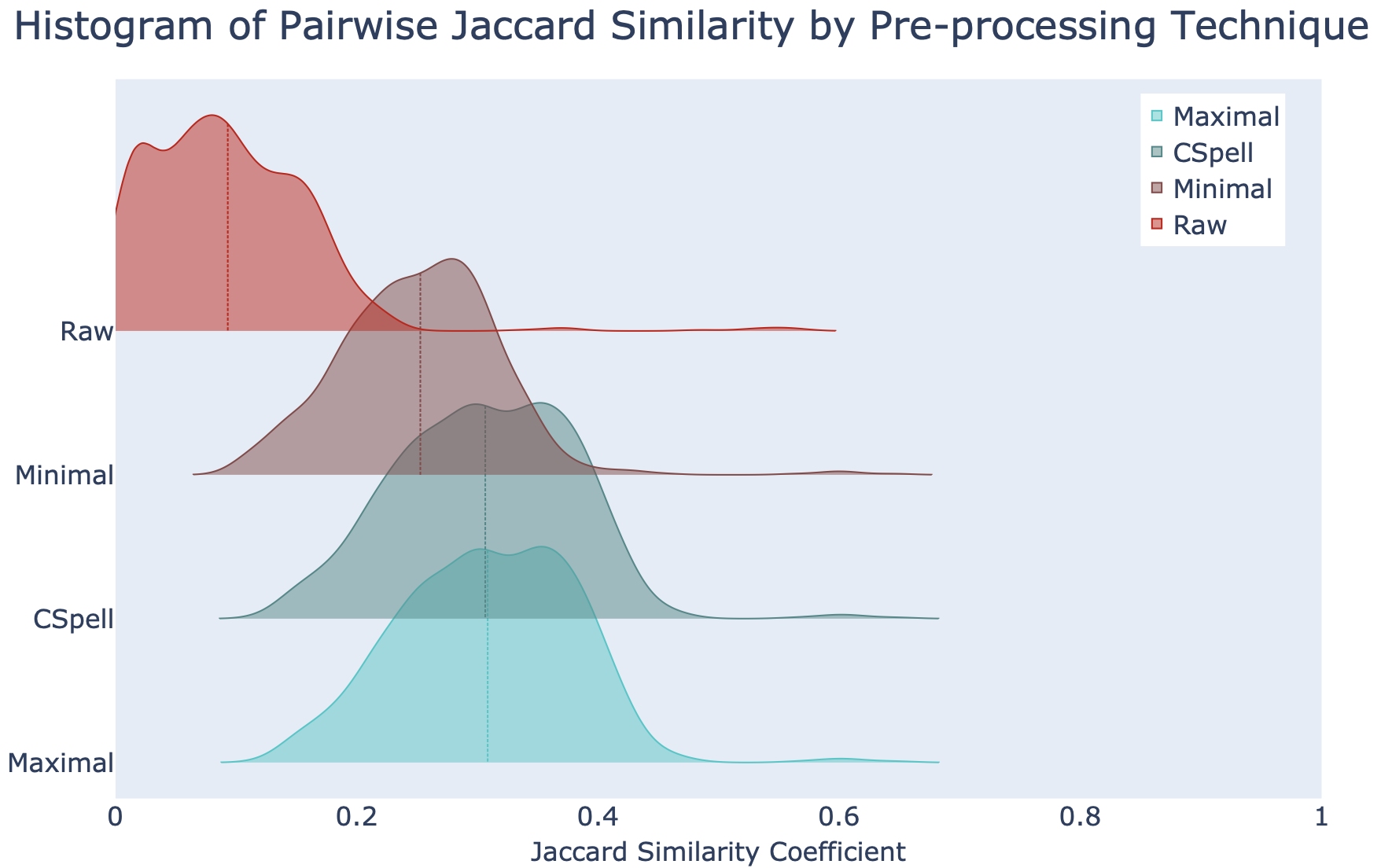


**Figure S4:** Composite Histogram of Jaccard Similarity between non-self institution pairs. Institution similarity is increased with each preprocessing technique.

**Figure S5:** F1-score vs. Jaccard Similarity and KLD. Each point represents an individual pair of non-self institutions. Blue dots are compared against Jaccard Similarity while red dots are compared against KLD. The minimal data preprocessing level was used in modeling.


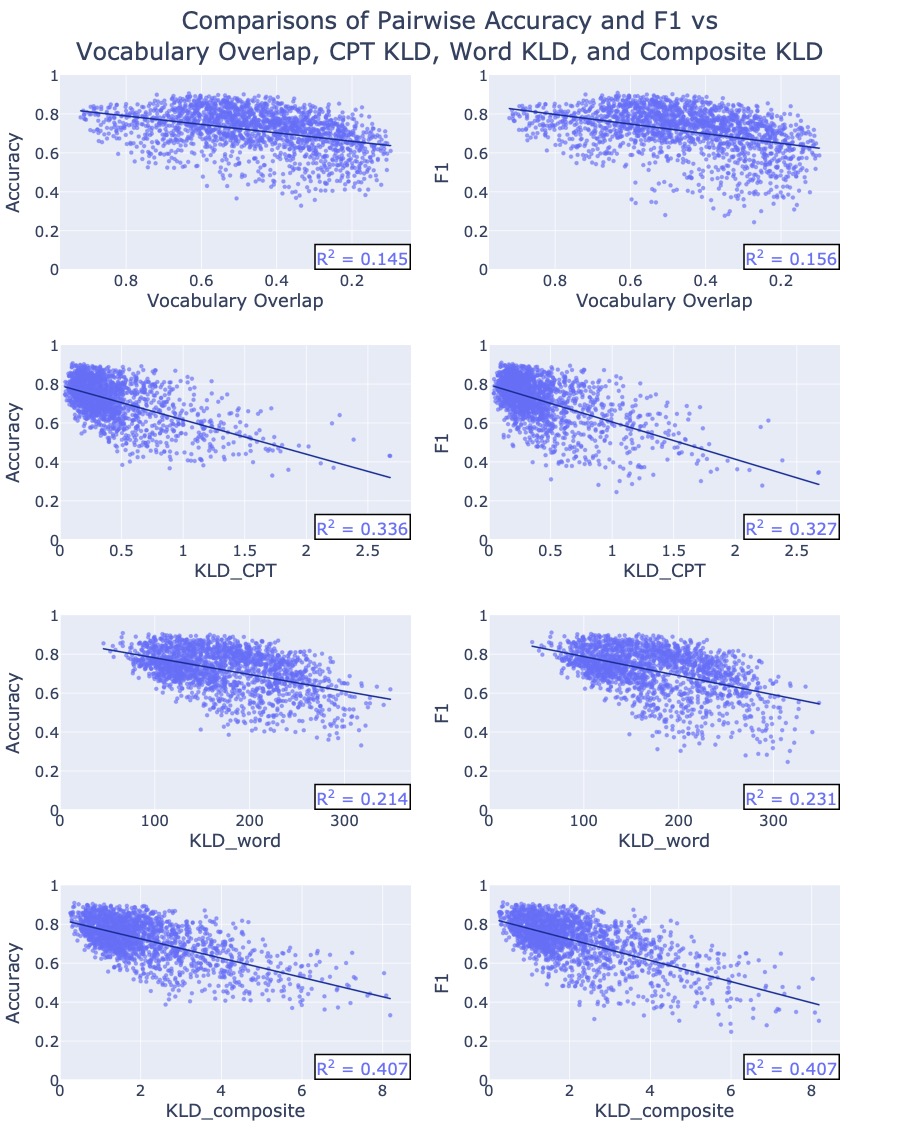


**Figure S6:** Accuracy and F1 vs. vocabulary overlap, KLD_CPT, KLD_Word, and KLD_Composite after minimal preprocessing. Each point of each subplot represents an individual pair of non-self institutions.
